# Developmental Trajectories of Loneliness Among Chinese Early Adolescents: The Roles of Early Peer Preference and Social Withdrawal

**DOI:** 10.3390/bs14111063

**Published:** 2024-11-07

**Authors:** Wanfen Chen, Bowen Xiao

**Affiliations:** 1 School of Education, Suzhou University of Science and Technology, Suzhou 215009, China; 2 Faculty of Education, The University of British Columbia, Vancouver, BC V6T 1Z4, Canada; bowen.xiao@ubc.ca

**Keywords:** loneliness, peer preference, unsociability, shyness, social withdrawal, developmental heterogeneity

## Abstract

This study aimed to examine distinct loneliness trajectories and to explore the roles of group-level peer preference and individual-level social withdrawal (i.e., unsociability and shyness) as predictors of these trajectories. Participants were 1134 Chinese elementary school students (Mage = 10.44 years; 565 boys). Data were collected from self-reports and peer nominations. Latent class growth analysis (LCGA) was employed to identify distinct trajectories of loneliness, and multinomial logistic regression was subsequently used to examine the relationships between these trajectories and their predictors. Results showed that three loneliness trajectories were identified: high increasing, moderate decreasing, and low decreasing. Participants at baseline with higher peer preference were more likely to belong to the low decreasing trajectory subgroup rather than the other two subgroups. Furthermore, those at Time 1 with higher unsociability had lower odds of being classified into the moderate or low decreasing trajectory subgroup compared to the high increasing trajectory subgroup. Additionally, participants at baseline with higher shyness had reduced likelihoods of following the low decreasing trajectory subgroup as opposed to the other two subgroups. These results have implications for how we understand both the different subgroups of loneliness trajectories and the predictions of peer preference and social withdrawal on these trajectories in Chinese early adolescents.

## 1. Introduction

Loneliness is commonly defined as a negative emotional response arising from the discrepancy between one’s ideal and actual perceptions of their interpersonal relationships [1]. Most people experience different levels of loneliness during their lifetimes [2]. Although temporary loneliness may be adaptive and normative, chronic loneliness poses a significant risk during the early years of life [3]. Chronic loneliness during childhood and adolescence has been linked to a range of psychological issues, including depressive symptoms, suicidal ideation, anxiety, and low self-esteem [4,5,6], as well as an increased risk for physical health problems, including poor general health, great sleep disturbance, morbidity, and mortality [4,7]. Therefore, it is essential to conduct longitudinal research to identify the factors that contribute to chronic loneliness among children and adolescents. However, existing studies in this field have primarily concentrated on Western societies [4,5]. To our knowledge, little research has examined distinct trajectories of loneliness in Chinese samples or explored predictors of these trajectories [8]. Thus, the current research aims to address these gaps by examining the developmental trajectories of loneliness among Chinese early adolescents, taking into account peer preference and social withdrawal factors such as unsociability and shyness.

### 1.1. Development of Loneliness from Childhood to Adolescence

Previous empirical research on the developmental trends of loneliness was inconsistent. For example, Heinrich and Gullone summarized that the prevalence of loneliness was the highest during adolescence [2]. However, Brennan suggested that there was no significant difference in loneliness incidences between childhood and adolescent age groups [9]. Additionally, Liu et al. claimed that Chinese children’s loneliness decreased over time from grade 2 to grade 5 [10]. Furthermore, a recent meta-analysis of longitudinal studies revealed that, in terms of mean-level development, loneliness tends to decrease throughout childhood and remains relatively stable from adolescence into the oldest old age [11].

These contradictory findings stem from comparisons based on mean-level loneliness, which assume that all individuals within the samples follow the same pattern of changes in loneliness. However, not all children and adolescents are able to successfully form and maintain satisfying social relationships to fulfill their need for belonging [12]. Therefore, individual differences may emerge in the development of loneliness, as not all children and adolescents experience the same patterns of social connection and support [6]. According to Bauer and Curran, researchers may not be able to obtain accurate and adequate information that appeared in distinct subgroups if developmental heterogeneity is not taken into account [13]. Indeed, Western studies have suggested that there existed subgroups in which individuals followed different developmental courses. For instance, Qualter et al. identified four loneliness trajectories in people aged 7–17 (data gathered at 2-year intervals), namely, a low stable (37% of the sample), a moderate decreasing (23%), a moderate increasing (18%), and a relatively high stable subgroup (22%) [4]. Schinka et al. found five discrete trajectories of loneliness (each wave at the age of 9, 11, and 15). These trajectory classes were referred to as low stable (49.1%), moderate increasing (31.6%), decreasing (10.7%), high increasing (4.5%), and chronic (4.1%) [5]. Additionally, Liu et al. found that Chinese children’s mean-level loneliness was initially low and decreased in a non-linear (i.e., quadratic) trend from grade 2 to grade 5 [10]. Given the differences in the sample’s age range (Mage = 10.44 years at wave 1) and the longitudinal intervals (once a year) in this study compared to previous research, it is reasonable to expect heterogeneity in the developmental trajectories of loneliness among Chinese early adolescents. We anticipate that most will follow a low, decreasing trajectory of loneliness. Additionally, we expect to observe moderate decreasing and high increasing loneliness trajectories as well.

### 1.2. Peer Preference and Developmental Trajectories of Loneliness

Peer preference refers to the extent to which a child is accepted and liked by their peer group [14]. Loneliness occurs when individuals perceive deficiencies in the quantity and/or quality of their social connections [11]. Peer relationships are among the most important social connections in early adolescence. Positive peer interactions significantly fulfill the need for security and belonging, both of which are essential for the development of socioemotional functioning, including the prevention of loneliness [15]. Moreover, Parkhurst and Hopmeyer’s developmental theory on the sources of loneliness suggests that a lack of prestige, popularity, and acceptance among peers can lead to feelings of loneliness in upper-grade elementary and middle school students [16].

Previous longitudinal studies have supported these theoretical perspectives, showing that children who are more popular among their peers tend to experience less loneliness [10,17]. For instance, Liu et al. reported that peer preference as a time-variant variable in latent growth curve modeling reduced loneliness among Grades 2–5 children [10]. In the research of Liu et al., results from the developmental cascade model indicated that peer preference could negatively predict children’s later loneliness [17]. Additionally, research on distinct trajectories of loneliness and its predictors among 7–17-year-old individuals showed that low peer preference was a predictor for highly stable and moderately increasing trajectories [4]. Taken together, guided by both theoretical and empirical evidence, we inferred that low peer preference serves as a risk factor for increasing loneliness during early adolescence.

### 1.3. Social Withdrawal and Developmental Trajectories of Loneliness

Socially withdrawn children and adolescents often remove themselves from peer interactions [18]. We proposed that two subtypes of social withdrawal—unsociability and shyness—might influence the developmental trajectories of loneliness. Unsociability refers to a non-fearful preference for solitude [19]. Unsociable individuals enjoy and value their alone time [20]. Nonetheless, researchers have suggested that unsociability may negatively impact the socioemotional development of older children and early adolescents [20,21]. Individuals who spend excessive time alone miss out on many opportunities for social interactions and skill development, which can negatively affect their social and emotional adjustment, leading to poor social skills, depressive symptoms, and loneliness [15,18]. Moreover, based on Maslow’s Hierarchy of Needs [22], humans have strong needs and motivations to form and maintain interpersonal relationships and to gain a sense of belonging, but unsociability may lead to a lack of social connections, which can cause emotional maladjustment such as loneliness [21,23].

Furthermore, researchers have suggested that cultural values influence the implications of different subtypes of social withdrawal [24]. Specifically, Chinese culture is a collectivist culture that focuses on maintaining harmony [25]. Thus, intentionally withdrawing from the social group is viewed as eccentric and abnormal within this cultural context [25]. Therefore, unsociability has been associated with a range of internalizing problems, particularly loneliness, within Chinese culture [21,24]. For instance, Bullock et al. [26] and Xiao et al. [21] both revealed that unsociability was positively related to loneliness in Chinese older children and early adolescents. Additionally, a short-term longitudinal study showed that unsociability perhaps acted as a risk factor for later loneliness among 10–14-year-old children [23]. Despite the consistently detrimental effects of unsociability on Chinese children, to our knowledge, no research has examined whether or how unsociability influences the trajectories of loneliness over time. Based on the aforementioned factors, we hypothesized that high levels of unsociability may serve as a risk factor for increasing loneliness over time among Chinese early adolescents.

Shyness is another subtype of social withdrawal, which refers to a temperamental trait characterized by anxiety and wariness in the face of social interaction [19]. In Western culture, shy children are perceived as deviant, immature, and incompetent [18]. From early childhood to adulthood, shyness is concurrently and predicatively linked to maladjustment [18], such as loneliness, depression, and peer difficulties [27,28]. As discussed before, in traditional Chinese society, being sensitive to others, wariness, and behavioral restraint are positively valued and viewed as social maturity and accomplishment [25]. In this cultural context, shy children may obtain support and encouragement from peers and adults, which in turn would help them develop socioemotional adjustment [25]. However, China has undergone massive economic reform and societal change over the past four decades. In the competitive and fast-paced urban environment, temperament and behavioral traits such as bravery, proactiveness, goal orientation, and expressiveness have become more adaptive for achieving progress and success [25]. This shift has led to a significant decrease in the adaptability of shyness. Indeed, recent research has shown that shyness in children and early adolescents is positively associated with internalizing problems, such as loneliness and depression, in contemporary urban China [28].

Specifically, recent cross-sectional and longitudinal studies have demonstrated that shy children and preadolescents are more likely to experience both concurrent and subsequent loneliness in suburban and urban Chinese areas [24,28]. For instance, Yang et al. indicated that Chinese children’s shyness had significant contributions to later internalizing problems (including loneliness) in a four-wave longitudinal study [28]. Therefore, we hypothesized that early adolescents with high levels of shyness are less likely to follow a decreasing trajectory of loneliness and are more likely to follow a high increasing trajectory of loneliness.

### 1.4. The Present Study

The aim of this study was to describe the developmental trajectories of loneliness and examine the differential effects of peer preference and social withdrawal (i.e., unsociability and shyness) on these trajectories among Chinese early adolescents. Gender was controlled for due to inconsistent findings in previous research regarding gender differences in loneliness trajectories. Based on the aforementioned literature, we hypothesized that the majority of participants would follow low or moderate decreasing loneliness trajectories while a minority would exhibit high increasing loneliness trajectories. Additionally, we posited that participants with high initial peer preference would be more likely to belong to the low decreasing trajectory group, whereas those with high initial levels of shyness would be more likely to follow a high increasing trajectory. Similarly, participants with high initial levels of unsociability were expected to have a higher probability of following the high increasing loneliness trajectory.

## 2. Materials and Methods

### 2.1. Participants

Participants were a sample of early adolescents from a large longitudinal study on the development of socioemotional functioning. They were recruited from 19 classes in five public elementary schools in Anhui Province, China. At Time 1 (T1), participants were 1134 fourth-grade students (Mage = 10.44 years, SD = 0.65 years; 565 boys).

Almost all participants in this sample identified their ethnicity as Han, which is the primary ethnic group (about 91% of the whole population) in China. Most of the subjects were from families with low socioeconomic status. Among their parents, about 28.71% of the fathers and 42.23% of the mothers received a junior high school or lower education, 64.77% of the fathers and 52.77% of the mothers received a high school education, and 6.52% of the fathers and 5.00% of the mothers received an education above high school.

Longitudinal data were annually gathered for two years in the same schools. At Time 2 (T2) and Time 3 (T3), 1045 and 1048 participants provided effective data, respectively. We utilized Little’s Missing Completely at Random test to examine missing data [29], with χ^2^ (106) = 130.365, *p* = 0.054, suggesting that there were no significant differences in main study variables between subjects who participated in all waves and those who did not.

### 2.2. Measures

#### 2.2.1. Loneliness

Participants rated their loneliness with a Chinese version of the self-report measure, revised by Asher et al. [30]. This single factor measure comprises 16 items (e.g., “Nobody talks to me”) employing a 5-point scale (1 meaning not true at all and 5 meaning always true). The mean score of the answers was computed, with higher scores meaning a higher degree of loneliness. This measure has been demonstrated to be valid and reliable in prior research in Chinese children and early adolescents [28]. The internal consistency reliabilities were 0.86 to 0.90 at T1-T3 in the current investigations.

#### 2.2.2. Peer Preference

Each participant nominated a maximum of 3 classmates with whom he/she preferred to be and 3 classmates with whom he/she preferred not to be (i.e., positive and negative nominations). As recommended by previous researchers [31], nominations were permitted for both same-gender and different-gender individuals. The number of nominations for each student received from every classmate was added up and subsequently standardized within each class to enable suitable comparisons. In line with previous research, an index of peer preference was calculated by subtracting negative nomination scores from positive nomination scores [31]. Higher scores on peer preference indicated higher peer likability in the class. This measure has been proven to be valid and reliable in Chinese children and adolescents [17,32].

#### 2.2.3. Unsociability

Unsociability was assessed by a peer nomination measure modified from the Revised Class Play [32,33]. In accordance with the prior procedure [33], each participant nominated up to three classmates who were most suitable for playing the specific role in a class play. Each specific role corresponds to one item. Four items were used to measure unsociability (e.g., “prefer to play alone rather than with others”). Subjects can nominate both same-gender and different-gender classmates. One’s nominations received from each classmate for each item were summed and then standardized within the class to account for differences in class size. Previous research demonstrated that this measure is a valid and reliable assessment of unsociability among Chinese children and adolescents [21]. In this research, the Alpha coefficient of this measure was 0.70 at T1.

#### 2.2.4. Shyness

The shyness of participants was assessed by a Chinese version of the Children’s Shyness Questionnaire [34], revised by Crozier [35]. This measure consists of 12 self-statements (e.g., “I feel shy when the teacher talks to me”) rated on a 3-point scale (1 = yes, 2 = sometimes, 3 = no). The example item and the other nine items were reverse-coded, after which the mean score of the responses was calculated. Higher scores indicated greater levels of shyness. In this study, the Alpha coefficient was 0.74 at T1.

### 2.3. Procedure

We annually administered a self-report measure of loneliness around the end of the academic year for 3 years. The data were collected in June 2022, June 2023, and June 2024. During data collection of this study, the participants studied and lived normally as they did before the COVID-19 pandemic. Additionally, at T1, we gathered data on peer acceptance rejection (for peer preference) and unsociability from peer nominations, and participants rated their own shyness. Participants were given the necessary explanations during the data collection. There was no indication that the participants encountered any challenges in comprehending the measures or procedures of this study. Ethical approval for the current study was obtained from the institutional review board of the first author’s affiliation. Informed consents were obtained from all subjects and their parents via the schools.

### 2.4. Statistical Analytic Strategy

Data analyses were performed with SPSS 23 and Mplus 7.2. Preliminary analysis was first performed, which mainly included descriptive statistics such as means, standard deviations, and correlation coefficients of the main study variables.

To test our primary hypothesis regarding the various developmental trajectories of loneliness and the predictive influence of peer preference and social withdrawal (i.e., unsociability and shyness) on these trajectories, we conducted the data analysis in three phases. Firstly, to examine the overall pattern in the trajectory of loneliness, we utilized latent growth curve modeling (LGCM) [36], which offers both mean level (referred to as intercept) and mean rate of change (referred to as slope). To evaluate model fit, we employed several statistical indicators, such as the chi-square index (χ^2^), in which smaller values are preferred, the Comparative Fit Index (CFI), which should exceed 0.90, and both the Standardized Root Mean Square Residual (SRMR) and the Root Mean Square Error of Approximation (RMSEA), which should be below 0.08 for an acceptable fit [37].

Secondly, to identify distinct classes of loneliness trajectories while accounting for gender differences, we utilized latent class growth analysis (LCGA) [38]. The LCGA method combines both variable-centered and person-centered approaches, allowing for the summarization of longitudinal data by modeling individual variability in developmental trajectories. It identifies a small number of trajectory classes based on differences between individuals (intercepts) and changes within individuals over time (slopes) [38]. Based on LGCM results, a series of LCGAs was performed, and model fit was evaluated using Bayesian Information Criteria (BIC), sample-size adjusted BIC (ABIC), Akaike Information Criteria (AIC), entropy, Lo–Mendell–Rubin likelihood ratio test (LMR-LRT), Vuong–Lo–Mendell–Rubin likelihood ratio test (VLMR-LRT), Bootstrapped LRT (BLRT), and practical usefulness [39]. Lower BIC, ABIC, and AIC values indicated better fit, while entropy values above 0.70 suggested good classification accuracy. LMR-LRT, VLMR-LRT, and BLRT tested whether models with more classes fit better, with BLRT being the most reliable [40]. Practical considerations, such as trajectory shape and class size (no less than 1%), were also factored into model selection [39].

Thirdly, after determining the optimal number of latent classes, we estimated the effects of the predictors (i.e., peer preference, unsociability, and shyness) on class membership using a multinomial logit model. One class was designated as the reference class, and the log odds ratios of belonging to the other classes were compared to this reference, allowing us to assess how each predictor distinguished class membership. Missing data were handled using the full information maximum likelihood method in Mplus 7.2.

## 3. Results

### 3.1. Preliminary Analysis

Means, standard deviations, and correlation coefficients for gender, loneliness, peer preference, unsociability, and shyness are shown in Table 1. We then conducted LGCM analyses to assess the mean-level trajectory of loneliness, with results displayed in Table 2 indicating an acceptable model fit. The general trajectory of loneliness showed a decreasing pattern over time. The significant variance in the intercept suggested that there was enough variability to justify exploring interindividual differences in loneliness trajectories using LCGA. Additionally, to determine whether there were any gender-based differences in the overall trend of loneliness, we performed a linear regression analysis of the intercept and slope of the loneliness trajectory on gender. The results showed that gender had no statistically significant effect on the intercept or slope of the loneliness trajectory (B = −0.03, 0.02, *p* > 0.1, respectively), indicating that the average loneliness trajectories for boys and girls were similar in this study.

### 3.2. Unconditional LCGA of Loneliness

As shown in Table 3, we estimated models with one to four classes, and the three-class model provided the best fit for the data. We selected the three-class model over the two-class model because it had lower BIC, ABIC, and AIC values, and the additional class represented a distinct group comprising 5.82% of the sample. The three-class model was preferred over the four-class model since the latter included a trajectory with only 0.53% of the sample, which reduced its practical usefulness. Furthermore, compared to the two- and four-class models, the three-class model offered the best balance of interpretability, model parsimony, and goodness-of-fit, as indicated by a significant and replicable BLRT value [41].

**Figure 1:**
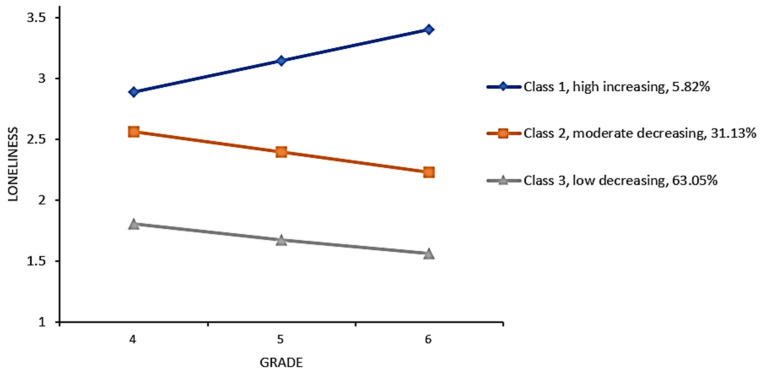
Estimated loneliness trajectories from the three-class LCGA model.

Three different trajectories of loneliness were illustrated, and the estimated means of the corresponding trajectories were marked in Figure 1. These trajectories included (1) a high increasing trajectory (intercept = 2.89, SE = 0.14, t = 21.03, *p* < 0.001; slope = 0.26, SE = 0.12, t = 2.20, *p* = 0.03; 66 students, 5.82% of the sample): this trajectory began with the highest level of loneliness in grade 4 and gradually increased throughout these three years; (2) a moderate decreasing trajectory (intercept = 2.56, SE = 0.06, t = 41.96, *p* < 0.001; slope = −0.17, SE = 0.04, t = −3.97, *p* < 0.001; 353 students, 31.13% of the sample): students following this trajectory presented a moderate initial level of loneliness in grade 4 and experienced a decrease within this duration; and (3) a low decreasing trajectory (intercept = 1.81, SE = 0.04, t = 46.14, *p* < 0.001; slope =−0.13, SE = 0.02, t = −6.47, *p* < 0.001; 715 students, 63.05% of the sample): students following this trajectory started with the lowest level of loneliness in grade 4, continued to decrease, and maintained the lowest level throughout the three-year span.

### 3.3. Conditional LCGA with Covariates Predicting Class Membership

We added covariates (i.e., peer preference, unsociability, and shyness) to the optimal model to examine potential differences among the three class memberships while controlling for gender. To obtain the coefficients of multinomial logistic regression for the predictors on latent class, the high increasing (HI) subgroup was set as a reference group (refer to Table 4). In comparison to the HI subgroup, early adolescents with higher initial levels of peer preference had higher likelihoods (1.73 times) of being categorized into the low decreasing (LD) subgroup, whereas those with higher initial unsociability were less likely to be classified into either the moderate decreasing (MD) subgroup (odds ratio, OR = 0.74) or LD subgroup (OR = 0.59). Additionally, early adolescents with higher initial shyness were significantly less likely to fall into the LD subgroup (OR = 0.11).

Furthermore, when the MD subgroup was assigned as the reference group, early adolescents with higher scores on initial peer preference had 1.55 times greater probabilities of belonging to the LD subgroup. Relative to the MD subgroup, early adolescents who had higher initial levels of shyness had a considerably lower probability of following the LD subgroup (OR = 0.15).

## 4. Discussion

Chronic loneliness has numerous negative effects on children’s and adolescents’ physical and mental health [2,3]. However, there is limited long-term longitudinal research on loneliness trajectories, particularly in non-Western contexts. Therefore, this study primarily focused on examining different trajectories of loneliness and exploring how early peer preference, unsociability, and shyness were related to these trajectories in a substantial sample of Chinese early adolescents.

### 4.1. Distinct Trajectories of Loneliness

In this study, three distinct loneliness trajectory classes were identified: low decreasing, moderate decreasing, and high increasing. These patterns are somewhat similar to those found in previous research, which also demonstrated developmental heterogeneity in loneliness during early adolescence [4,5,7].

Results revealed that most of the participants followed a low decreasing (63.05%) or moderate decreasing (31.13%) trajectory of loneliness. These findings were partly in line with previous studies that reported large ratios of low stable or moderate decreasing subgroups (37%, 23%, respectively; 49.1%, 10.7%, respectively; 81.5% in low stable subgroup) in children and adolescents [4,5,8]. Notably, in this study, the low level of loneliness was not stable but gradually decreased over the investigation period. Additionally, the two decreasing trajectories began at different initial levels, which aligned with the mean-level changes in loneliness over time. Peer groups play a critical role in providing children and early adolescents with a sense of belonging and intimacy [10,16]. In the upper grades of primary school, older children and early adolescents begin to place greater importance on their acceptance by peers, along with their status and reputation within peer groups [16]. However, the formation and consolidation of peer groups is a gradual process. During the initial stages of forming stable peer groups, individuals encounter various changes and uncertainties, which can increase their likelihood of experiencing loneliness [10]. As the group structure gradually stabilizes, individuals begin to integrate into the group and adapt to the social dynamics. As a result, their feelings of loneliness may decrease over time [10].

Finally, a minority of individuals (5.82% of the sample) in this study were in the high increasing subgroup. This result was in line with previous loneliness studies [4,5] and might be associated with one’s social competence. If early adolescents consistently lack a sense of belonging, they may experience chronic or increasing loneliness, putting them at risk for a variety of problems, including emotional, social, psychological, and physical issues [4,5,6,7].

### 4.2. Predictive Associations Between Peer Preference and Loneliness Trajectories

Peer preference promotes a high sense of popularity and prestige among peers [2,16], which can act as a protective factor against loneliness in early adolescents. In line with the previous study [4], the current research discovered that, generally, early adolescents getting higher scores on initial peer preference were more inclined to belong to the low decreasing subgroup compared to the other two subgroups. Early adolescents who initially displayed high levels of peer preference were less likely to experience loneliness following a moderate decreasing or high increasing trajectory. These findings suggest that, in Chinese culture, early peer preference is a key predictor of loneliness trajectory class membership among early adolescents. Being highly accepted and preferred by peers helps early adolescents fulfill their need for belonging and desire for interpersonal attachments [12,22], which may prevent or reduce loneliness over time.

### 4.3. Predictive Associations Between Social Withdrawal and Loneliness Trajectories

Consistent with the research on solitary activities and loneliness trajectories among Western children and early adolescents [4], our findings revealed that Chinese early adolescents with high initial levels of unsociability were more likely to belong to the high increasing loneliness trajectory rather than the other two classes. This suggests that unsociability at an early developmental stage is a key predictor for the development of loneliness. Children and adolescents who intentionally withdraw from their peer groups and spend excessive time alone are likely to experience a lack of social connectedness and a diminished sense of belonging [21]. However, within the context of Chinese culture, group orientation and social connectedness are traditionally emphasized and continue to be highly valued in recent years [25]. In fact, Chinese children are encouraged and supported by their mothers to demonstrate social connectedness and attachment from a very young age [42]. Taken together, it is not surprising that early adolescents’ initial unsociability may lead to high and increasing levels of loneliness over time.

Additionally, our results revealed that early adolescents with higher initial shyness were less likely to be classified into the low decreasing loneliness trajectory and more likely to belong to the moderate decreasing or high increasing subgroups. These findings suggest that early shyness is a significant predictor of loneliness trajectories. This aligns with recent research showing a positive relationship between shyness and loneliness among Chinese children and early adolescents [24]. Researchers also found that shyness can positively predict loneliness one year later in Chinese children and early adolescents [28]. Shy early adolescents often lack social skills due to their fear or wariness of challenging social situations [41], and this can lead to the development of more negative self-perceptions. As a result, they may experience increasing loneliness over time.

### 4.4. Limitations and Future Directions

It is important to acknowledge some limitations of this research. First, the study was conducted with a sample of Chinese early adolescents, and given the specific historical and cultural context, caution is necessary when extrapolating the findings to other societies. Additionally, China exhibits significant regional differences in social, economic, and cultural development. Therefore, researchers should be cautious when generalizing the results to other regions of China, including major urban centers or remote rural areas. Future studies should consider cross-cultural comparisons (e.g., between Chinese and Western societies) and cross-regional studies (e.g., urban vs. remote rural areas in China) to provide a broader understanding of these issues.

Second, this research only revealed how peer preference and social withdrawal predicted loneliness trajectories. Given that previous studies have shown that parent–child relationships [8,43], teacher–child relationships [8,44], and peer relationships, such as peer victimization [45] and friendship quality [26], have impacts on loneliness, it would be intriguing to explore the relationships between these factors and loneliness in future research. Additionally, intrapersonal and interpersonal factors may interact throughout development, influencing loneliness trajectories [8,15]. Future researchers may find it particularly interesting to investigate how these factors interplay in predicting the developmental trajectories of loneliness over time.

Third, this study did not examine how changes in loneliness affect psychological and physical health during early adolescence. Although previous research has suggested that individuals on high stable or increasing loneliness trajectories tend to report more socioemotional problems, such as depressive symptoms, anxiety, social skills deficits, and suicidal ideation [7], these findings have yet to be explored in a non-Western context. Future research could explore how these health indicators vary across different developmental trajectories of loneliness, which may help us gain a deeper understanding of the impact of loneliness on psychological and physical well-being.

Finally, one possible limitation of the present study is the potential conceptual overlap among the measurement dimensions. Although the constructs of peer preference, unsociability, and shyness were treated as distinct predictors of loneliness trajectories, these dimensions may share some conceptual similarities, potentially influencing the interpretation of our findings. Future research should consider using more refined measures or conducting factor analyses to better distinguish these dimensions.

### 4.5. Strengths and Implications

The present study integrated person-centered and variable-centered analyses in a large sample to identify distinct developmental trajectories of loneliness among early adolescents in a Chinese cultural context. In addition to the overall decrease in loneliness over time, the study revealed substantial heterogeneity in these trajectories. Three developmental trajectory classes were identified, and they were significantly predicted by peer preference, unsociability, and shyness in different ways. These findings underscore the importance of applying a combination of variable-centered and person-centered approaches to study the development of loneliness.

Moreover, the current study has important implications for designing effective prevention and intervention strategies for early adolescents vulnerable to chronic or increasing loneliness. First, researchers and educators should place greater emphasis on identifying the high increasing loneliness subgroup and encourage their participation in interventions that address not only loneliness but also other aspects of psychosocial maladjustment. Such interventions can have broad benefits, as highlighted in meta-analyses of loneliness interventions [46]. Second, implementing social skills training programs for early adolescents with low peer preference and/or high social withdrawal may reduce the likelihood of them following a high increasing loneliness trajectory. Third, schools and teachers should create an inclusive classroom climate and culture to promote peer interaction that meets students’ need for relatedness [47]. Meanwhile, incorporating evidence-based social and emotional learning (SEL) programs into standard educational curricula may significantly improve students’ social and emotional skills, attitudes, and behaviors, which would be helpful for reducing social difficulties and loneliness among early adolescents [48].

## Figures and Tables

**Table 1:** Correlation coefficients, means, and standard deviations of main study variables.

	1	2	3	4	5	6	7
1. Gender	1						
2. Loneliness G4	−0.00	1					
3. Loneliness G5	−0.05	0.50 **	1				
4. Loneliness G6	0.02	0.43 **	0.48 **	1			
5. Peer preference G4	0.09 **	−0.25 **	−0.22 **	−.022 **	1		
6. Unsociability G4	−0.04	0.18 **	0.19 **	0.19 **	−0.32 **	1	
7. Shyness G4	0.08 *	0.31 **	0.22 **	0.17 **	−0.03	0.05	1
*Mean*	1.50	2.13	1.98	1.89	0.02	−0.01	1.82
*Standard deviations*	0.50	0.66	0.66	0.67	1.51	0.98	0.37

Note: Gender, 1 = boys, 2 = girls; G, grade; * *p* < 0.05. ** *p* < 0.01.

**Table 2:** Model fit, means, and variances of intercept and slope in LGCM of loneliness.

Model	Model Fit					Mean		Variance	
	*χ*^2^ (*df*)	*p*	CFI	RMSEA	SRMR	Intercept	Slope	Intercept	Slope
LGCM	3.38 (11)	0.066	0.99	0.05	0.01	2.13 ***	−0.12 ***	0.25 ***	0.03 *^+^*

Note: LGCM, latent growth curve model; + *p* < 0.1. *** *p* < 0.001.

**Table 3:** Results of different latent class growth analyses (LCGA) of loneliness.

Class	BIC	ABIC	AIC	Entropy	LMR-LRT	VLMR-LRT	BLRT	Class Probability
1	6192.285	6176.404	6167.118	N/A	N/A	N/A	N/A	1
2	5640.996	5615.586	5600.728	0.747	<0.001	<0.001	<0.001	0.749/0.251
3	5529.961	5495.022	5474.593	0.732	0.010	0.008	<0.001	0.631/0.058/0.311
4	5502.984	5458.516	5432.515	0.762	0.004	0.003	<0.001	0.005/0.605/0.320/0.070

Note: N, 1134; BIC, Bayesian information criterion; ABIC, sample-size adjusted BIC; AIC, Akaike information criterion; LMR-LRT, Lo–Mendell–Rubin likelihood ratio test; VLMR-LRT, Vuong–Lo–Mendell–Rubin likelihood ratio test; BLRT, bootstrap likelihood ratio test; N/A, Not Available.

**Table 4:** Multinomial logistic analyses of the relationships between loneliness trajectory class membership and covariates.

Covariate	Moderate Decreasing			Low Decreasing			Low Decreasing		
	vs. High Increasing						vs. Moderate Decreasing		
	Est.	*S.E.*	*OR*	Est.	*S.E.*	*OR*	Est.	*S.E.*	*OR*
Gender	0.04	0.35	1.04	−0.02	0.33	0.98	−0.06	0.21	0.94
Peer Preference	0.11	0.12	1.11	0.55 ***	0.13	1.73	0.44 ***	0.08	1.55
Unsociability	−0.30 *	0.12	0.74	−0.53 **	0.16	0.59	−0.23	0.14	0.79
Shyness	−0.32	0.50	0.73	−2.25 ***	0.48	0.11	−1.93 ***	0.30	0.15

Note: Est., estimate; S.E., standard error; OR, odds ratio. All values are unstandardized estimates. * *p* < 0.05. ** *p* < 0.01. *** *p* < 0.001.

## Data Availability

The datasets involved in the present study are available from the corresponding author upon reasonable request.
